# American Practitioner

**Published:** 1871-12

**Authors:** 


*American Practitioner.*

We have to acknowledge the receipt of elegantly bound volumes No. III and IV, 1871, of the *American Practitioner*, edited by David W. Yandell, M. D., Professor of Clinic Surgery in the University of Louisville, and Theophilus Parvin, M. D., Professor of Medical and Surgical Diseases of Women, in the same university. This journal is one of the most attractive and instructing medical journals of the country, being conducted with great ability and discrimination. It is published monthly in Louisville and makes two yearly volumes of near four hundred pages each. We extend to the editors our hearty thanks and congratulations, and assure them that their volumes will be highly prized, containing, as they do, a faithful record of the progress of medicine and surgery.

